# A proof-of-principle study of multi-site real-time functional imaging at 3T and 7T: Implementation and validation

**DOI:** 10.1038/srep08413

**Published:** 2015-02-12

**Authors:** Sebastian Baecke, Ralf Lützkendorf, Johannes Mallow, Michael Luchtmann, Claus Tempelmann, Jörg Stadler, Johannes Bernarding

**Affiliations:** 1 Institute for Biometry and Medical Informatics, Otto-von-Guericke-University Magdeburg; 2 Department of Neurosurgery, Otto-von-Guericke-University Magdeburg; 3 Department of Neurology, Otto-von-Guericke-University Magdeburg; 4 Leibniz Institute for Neurobiology Magdeburg

## Abstract

Real-time functional Magnetic Resonance Imaging (rtfMRI) is used mainly for neurofeedback or for brain-computer interfaces (BCI). But multi-site rtfMRI could in fact help in the application of new interactive paradigms such as the monitoring of mutual information flow or the controlling of objects in shared virtual environments. For that reason, a previously developed framework that provided an integrated control and data analysis of rtfMRI experiments was extended to enable multi-site rtfMRI. Important new components included a data exchange platform for analyzing the data of both MR scanners independently and/or jointly. Information related to brain activation can be displayed separately or in a shared view. However, a signal calibration procedure had to be developed and integrated in order to permit the connecting of sites that had different hardware and to account for different inter-individual brain activation levels. The framework was successfully validated in a proof-of-principle study with twelve volunteers. Thus the overall concept, the calibration of grossly differing signals, and BCI functionality on each site proved to work as required. To model interactions between brains in real-time, more complex rules utilizing mutual activation patterns could easily be implemented to allow for new kinds of social fMRI experiments.

Real-time functional magnetic resonance imaging (rtfMRI) has recently become the subject of increased interest primarily due to its use for brain-computer interfaces (BCIs) and neurofeedback techniques. In MR-based neurofeedback experiments, volunteers receive information about activation of specific areas of their brains in order to record their ability to regulate those areas based on the feedback they receive^1^,^2^,^3^,^4^. This information is provided in real-time by an online statistical analysis of change in the so-called BOLD (blood oxygen level dependent) signal, which serves as an indirect measure of neuronal activation. Because of the underlying physiological processes, real-time fMRI has a natural delay of several seconds, but despite this limitation, the spatial resolution is excellent and thus enables detection of activation even in subcortical structures such as the hippocampus or the amygdala. In addition to being used in neurofeedback, rtfMRI was recently used to monitor online the internal mental states that encode for decision making^5^ or bi-stable perceptions^6^. Real-time fMRI can also be used by a subject to communicate with other humans using interfaces to machines or controlling devices such as protheses^7^,^8^. This application is called a brain-computer interface (BCI), a process that was established earlier using EEG techniques^9^,^10^,^11^. One important goal of many experiments in the area of BCIs is to develop neuroprosthetics for patients with severe illnesses such as locked-in syndrome^7^,^8^.

But until now, rtfMRI experiments have been restricted to one volunteer at a time lying in a scanner. Interestingly, EEG (electroencephalography)^10^,^11^,^12^, and more recently fNIRS (functional near-infrared spectroscopy)^13^,^14^,^15^, have been successfully employed for real-time experiments of coupled brains. Though there were a small number of fMRI experiments that were realized by scanning two volunteers at the same time, the data acquired in those experiments had to be analyzed offline^16^,^17^,^18^,^19^,^20^,^21^. The main goal of these few studies was the analysis of brain activation during the interaction of the volunteers, which is an important research field for social fMRI or neuro-economic studies. However, fMRI studies that investigate social processes between interacting partners are in most cases conducted under conditions where subjects are seldom in direct contact with each other. Rather, they react to or interact with stimuli presented in a fixed context by another participant or even a computer. Data are then evaluated offline to analyze activated brain networks^22^,^23^,^24^,^25^. But designing experiments to examine the full potential of dynamic interactions (e.g., subjects adapting their own strategies when they receive specific signals from partners) requires a different approach. The main prerequisite for that is the analysis of data in real-time and the subsequent immediate use of those results within the experiment.

So far, the only simultaneous, multi-site rtfMRI experiments to have been reported is the single conference contribution by Goebel et al.^26^ (as well as some preliminary results of this study from our own group^27^,^28^). The main obstacles to this construct are surely the extensive technical effort involved in connecting two or more scanners, the guarantee that BOLD signals can be compared between several volunteers, the exchange of analyzed data during ongoing measurement, and the triggering of dynamic events according to simultaneous complex information from different volunteers.

Thus the goal of our study was to establish a flexible environment for connecting scanners, even those in remote locations and with differing hardware features, in order to allow more researchers to realize new classes of experiments when monitoring interacting brains in real-time. Multi-site rtfMRI could allow us to investigate new paradigms, such as changes of strategy in mutual interactions or controlling objects in shared virtual environments. However, the differing features of each scanner's hardware and the variations in inter-individual brain activation levels required a new strategy in order for us to be able to compare acquired signals.

To that end, we adapted a previously developed framework^29^ in order to allow for multi-site rtfMRI, not just one fixed configuration. We also wanted to establish a flexible system that would allow for varying experimental settings in which we could include more scanners and controlling objects independently or jointly. Results were displayed separately or in a shared view to each participant. Rules for allowing or disallowing mutual interaction were implemented in separated modules to enable easy and quick adaption to changing experimental conditions.

The new framework also had to be designed to support connections of remote scanners via internet (direct connections were not considered to be a forward-looking solution) since only this internet connection would allow wider application (sites with several adjacent scanners are quite rare). This in turn required integrating a signal calibration procedure because scanners at different sites would normally exhibit varying signal properties like a difference in field strengths, coil characteristics, or other machine-dependent factors. The experimental design also had to allow for differing abilities in volunteers to reach a certain percentage of brain activation, especially when different field strengths are involved.

Here we report on the concept and its validation in a proof-of-principle study with twelve volunteers (six each on 3T and 7T). A well-known motor paradigm had to be performed on both scanners. Subjects can much better control their motor actions and voluntarily reproduce them than they can regulate their emotional brain areas. Prior to the main experiment, brain activation of each volunteer was analyzed in real-time, and the results were used to calibrate the signal on each site with respect to the maximum achievable individual brain activation (calibration experiment). To validate this calibration procedure, we hypothesized that the outcome of the subsequent main experiment, in which the motor activity level had to be varied in order to put a virtual object at predefined locations on the screen, would be the same at both sites. To test the full functionality of the framework, three different tasks were measured in the main experiment. The first task - without any information exchange between participants—validated the calibration procedure, while two other tasks included information exchange between partners and a mutual dependence of each on the performance of the partner for success. The focus of this report is the validation of the concept and the calibration procedure, that is, the experiment without mutual information flow and dependencies. A more extended analysis of influences of information flow and mutual dependencies will be presented in a forthcoming publication.

## Results

The new extended framework was successfully implemented. The overall workflow of the calibration procedure and the main measurement ran as required by the concept. The individual cortical activations from the motor task were determined in real-time when running the functional localizer and the results were successfully used for calibrating the activity of each volunteer for the subsequent main measurement. In the main experiment, subjects were able to control the position of an object by varying the activation strength of their sensorimotor cortex (SMC). In the section of the experiment that involved no mutual information flow, 57% of all blocks were successfully completed. In the section that did involve mutual flow of information, the success rate dropped to an average of 36%.

**Figure 1:**
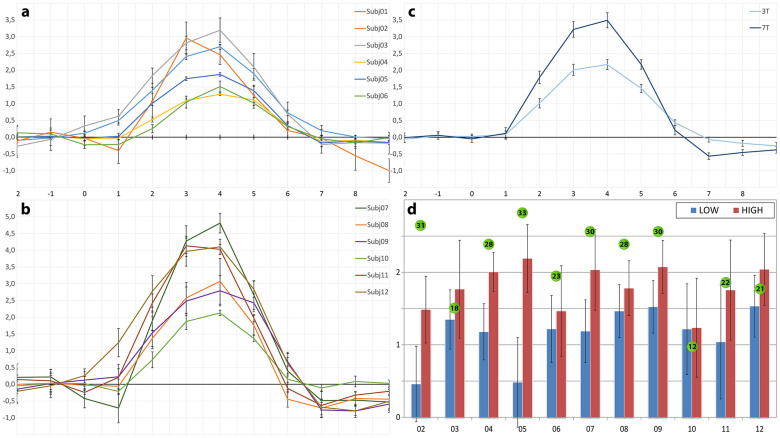
(a–c) Results of the functional localizer: Individual and mean BOLD signals of the 3T and 7T group. (a) 3T single subject BOLD signals from the functional localizer with standard error. Max. amplitude: subj01 = 2.69%, subj02 = 2.95%, subj03 = 3.19%, subj04 = 1.28%, subj05 = 1.87%, subj06 = 1.51%; (b) 7T single subject BOLD signals from the functional localizer with standard error. Max. amplitude: subj07 = 4.81%, subj08 = 3.07%, subj09 = 2.79%, subj10 = 2.12%, subj11 = 4.13%, subj12 = 4.10%; (c) Mean BOLD signals from the functional localizer for 3T and 7T with standard deviation. Max. amplitude: 3T = 2.17%, 7T = 3.48%. (d) Maximum amplitudes in the low and high sensorimotor activation at 3T and 7T in the main experiment, averaged over all three tasks. The bars represent the mean percentage increase of the BOLD signals with the according standard errors. The number in the green circle represents the total number of successful runs. Subject 1 was excluded from the analysis, subjects 2–6 were measured at 3T, subjects 7–12 were measured at 7T. A highly significant difference between weak and strong tapping was found.

As we had expected, the off-line analysis of the calibration experiment revealed that the amplitudes of the hemodynamic response of the contra-lateral sensorimotor cortex varied to a certain degree within each group (Figures 1a and b). While the mean hemodynamic response functions (HRF) when averaged among the volunteers of each group were about 60% higher for 7T than for 3T (Figure 1c), the amplitudes of three individual responses in the 7T group were in the range of the HRF amplitudes of the 3T group. This demonstrates that a direct comparison of activation amplitudes between different volunteers may be difficult to effect or may produce misleading results.

**Figure 2:**
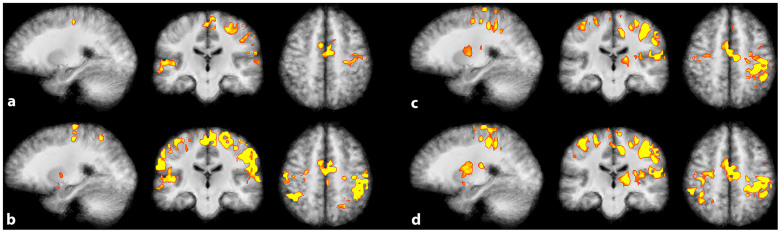
Activation patterns as a function of motor execution in the main experiment. Statistical maps (p < 0.001 FDR corrected, cluster threshold 30 voxels) of two representative volunteers overlaid on the anatomical group average. (a) 3T single-subject analysis – weak tapping task; b) 3T single-subject analysis – strong tapping task; (c) 7T single-subject analysis – weak tapping task; (d) 7T single-subject analysis – strong tapping task.

The differences for each subject for the two activation levels in the main experiment are shown in Figure 1d. These differences were further analyzed to ensure that the calibrated signals at different magnetic field strengths led to comparable results. Using a mixed model to allow for correlation of the data, we found a highly significant difference (p < 0.0001) between weak and strong tapping (Figure 1d). Typical changes in activation patterns related to the strong and weak tapping task for two volunteers can be seen in Figure 2.

The percentage of successfully performed tasks per volunteer of 3T and 7T group members has been compared using an ANOVA for repeated measurements, with the field strength as a between-subject factor and the three tasks as a within-subject factor. There was no overall effect for field strength (p = 0.514), but a strong effect for tasks (p = 0.001, Greenhouse-Geisser correction), without interaction between the two factors (p = 0.348, Greenhouse-Geisser correction). Thus, there is no advantage determining success for either one of the groups. All statistical analyses were conducted using SAS (SAS/STAT® 9.2, SAS Institute Inc.).

**Figure 3:**
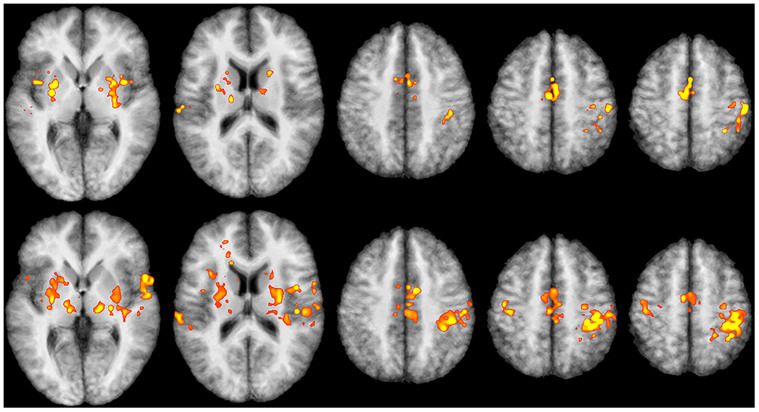
Representative slices of the random effects analysis of the group data for both tapping conditions superimposed on the averaged T1-weighted images of all eleven subjects (radiologic convention). The slices display the main parts of the sensorimotor system (primary contralateral and ipsilateral SMC, bilateral basal ganglia and supplementary motor area (SMA). Upper row: 3T (without subject 1 for details s. text), p < 0.005, FDR corrected. Lower row: 7T, p < 0.005, FDR corr. (cerebellum was not scanned at 7T). For a detailed cluster description, see Table 1.

The well-known typical activation patterns of standard finger-tapping paradigms can be clearly detected here. Besides the overall increase of activation in many parts of the cortical sensorimotor network (contralateral and ipsilateral SMC, supplementary motor area [SMA]), there was increased activation in other areas, such as the putamen and the thalamus, that was also clearly detectable with a high statistical significance (p < 0.005, FDR corrected) (see Table 1 and Figure 3). The activation was more pronounced at 7T in both contra- and ipsilateral SMC and bilaterally in the thalamus, while SMA and both putamen exhibited a somewhat greater activation at 3T.

## Discussion

In this proof-of-principle study, the implementation of the concept of performing multi-site, real-time fMRI on scanners with different field strengths was successfully realized and validated. Using a simple but reliably controllable and reproducible motor task, we could distinguish both activation levels with the implemented calibration and signal detection framework. With the use of relative rather than absolute signal changes, the modulation of the motor cortex activation on both sites could be processed in a unified manner to successfully manipulate the virtual object on each site. Since the position of the virtual object served as a visual feedback for control of a specific brain region (here the SMC), the main prerequisite for establishing a multi-site neurofeedback tool was also fulfilled.

The validation of the concept only needed a comparison of both groups separately with respect to the factors of each local site. In the results from that comparison, no significant differences were found between the 3T and the 7T groups. However, one cannot fully exclude the possibility that differences may be detectable in the examination of larger groups in environments involving ultra-high field scanners (see below).

The scanning of two people at the same time was already achieved some time ago^16^,^17^, but those studies used scanners with the same field strengths and without correcting for potential remaining hardware-dependent differences on local sites. The applications were therefore constrained to a few sites that had access to or possessed scanners with the same technical features. Additionally, in most of those experiments, data were not analyzed in real-time and individual activation differences were only taken into account in the post-experiment statistical analysis. Exchange of information occurred not with respect to brain activation but with respect to the results of the tasks. For example, Fliessbach et al. transferred the decisions made by one partner to the other partner^17^.

Our experimental setup provides features not reported until now that may allow a larger community to perform new classes of experiments by connecting several scanners: (1) the successful realization of multi-site real-time fMRI analysis; (2) the use of relative instead of absolute signals, which can take into account different scanner characteristics and different individual brain activation during the same task; (3) an internet-based concept that allows one to connect scanners even if they are distant from each other or located in separated firewall-secured networks; (4) the extension of unified experiment control and data analysis in real-time^30^ to include multi-site real-time fMRI; and (5) a shared virtual platform integrated into the framework that allows experiments to be conducted with real-time exchange of information or mutual interaction.

But anyone planning to implement this kind of challenging, multi-site infrastructure needs to consider several points. Connecting two (or even more) scanners is quite demanding if the MR-scanners are located in medical or medical-related research environments where data-protection requirements are very restrictive. Another problem may arise even with scanners that have the same field strengths, because elements of the technical environment (shims, coils, hardware specifications, sequence parameters, etc.) will always lead to somewhat different MR signals, even if the same person, positioned successively in different scanners, is exhibiting the same physiological activation in each. This discrepancy requires a calibration procedure ensuring the comparability of signals, which, however, seems not to have been reported so far. Our results showed that using calibrated relative signal changes instead of absolute signals allows a direct comparison of the 3T group and the 7T group, at the same time accounting for individual differences in brain activation. No significant differences in the number of successful runs were found between the two groups. Thus comparing relative signal changes makes multi-site rtfMRI feasible even in a heterogeneous environment with grossly differing BOLD signals. Despite the successful calibration procedure, though, additional aspects have to be taken into account when comparing BOLD signals from high-field and ultra-high-field scanners, especially when going from 3T to 7T. Many factors have to be considered that may alter or counteract the expected signal increase and detectable activation patterns: the increased field inhomogeneity at 7T; varying coil architectures and reconstruction algorithms; resolution and limitations due to increased specific absorption rate (SAR); geometric distortions; and signal losses due to increased susceptibility artefacts and decreased T2* relaxation^31^. In addition to these rather technical factors, increased static and physiologic noise contributions^31^ (mainly pulsatile-induced motion artefacts) and severe susceptibility-induced signal losses (especially when scanning frontal, caudal or inferior temporal brain regions) have to be taken into account, specifically when trying to monitor or modulate emotion-related brain structures such as the nucleus accumbens or the amygdala. It has been demonstrated that physiological noise can be corrected retrospectively at 7T^32^, but real-time implementation will require additional adaption of the concept. All factors may reduce statistical significance and thus counteract to some degree the signal enhancement gained at 7T. While the signal at 7T is still considerably higher than at 3T (Figures 1–3) for the sensorimotor cortex (where geometric distortions are negligible using standard EPI sequences with phased-array coils and parallel imaging), subcortical structures may exhibit greater signal losses due to increases in susceptibility and pulsatile motion artefacts. This difference can be seen in Table 1, where basal ganglia exhibit comparable or somewhat higher T-values at 3T than at 7T, yet the number of activated voxels is still greater at 7T, except for the right thalamus and the putamen. Another point to take into consideration is the increased SAR at higher field strength. But reducing the flip angle may still allow to acquire more slices or to increase the resolution while remaining below the SAR limit. At 3T, this strategy was shown to yield good results for fMRI experiments^33^, but the accompanying optimization of sequence parameters has yet to be determined for 7T. Similar arguments apply for the resolution, as discussed in Olman & Yacoub^31^. In our experiment, we wanted to compare similar resolutions between 3T and 7T even though the 7T signal may have been degraded to some degree due to de-phasing within the 4 mm slice. However, increasing the resolution increases the time required for reconstruction (especially when using phased-array coils with 24 or more elements) and data analysis. For our proof-of-principle study, data acquisition and processing time was well within TR of 2 s for a 64 × 64 matrix and 31 slices (3T). Higher in-plane resolution would require fewer slices or applying faster algorithms as well as using high-performance computing (e.g., provided by GPU cluster)^34^. Optimizing sequences, like using 3D instead of 2D EPI sequences, might also prove advantageous^35^.

A more difficult problem when comparing spatial distribution of activation is the BOLD signal originating at different field strengths. At 3T, the BOLD signal results mainly from larger draining veins, thus being mostly of intravascular origin, while at increased field strengths, the T2 relaxation time of blood decreases, thereby leading to increased extravascular susceptibility effects around vessels^36^. This effect may be important since vascular architecture of the brain usually differs from individual to individual as well as from region to region within the same brain.

Motion artefacts are another major obstacle in real-time fMRI. However, activation is usually displayed in real-time to the experiment's supervisor, who can then check the data quality during the experiment, thus allowing for a repeat of measurement if increased noise or artificial signals resulting from increased motion are detected online (e.g., on the borders of the brain and cerebrospinal fluid).

In addition to these technical and physiological aspects, some perception-related and cognitive factors have to be considered. Our results show that, despite a subject's good voluntary control of motor activation, a certain variability of the signals in the SMC remained (Figure 1). An explanation for this result may be the subject's loss of attention and concentration (the experiment requires a very high level of concentration to maintain a constant tapping strength reproducible with small variances. Although repetitive and simple tasks may increase the risk of attention loss, they may also serve to check to what degree a person undergoing a real-time experiment may be able to keep a focused attention and concentrate at a high level as well as to recruit the required motor memory and planning aspects. In a multi-person experiment, the attention loss of one participant may affect that participant's partner, thus decreasing the validity of the experiment. It may therefore be advantageous to develop methods for determining online the degree of attention being exhibited. In a multi-person experiment, mutual information flow or the dependence of one performance on another may affect the outcome of an experiment. In our experimental design, for example, if one subject reached the target position (which would be a success as a single participant) but the partner missed the goal, that run was deemed unsuccessful (e.g., in the cooperation condition). A loss solely due to the partner's performance may in fact affect the motivation of the subject. We have observed drops from 57% success in single runs to 36% success in social runs, which may reflect that specific influence. Future experiments will have to analyze these factors in more detail, but it seems evident that in light of them, initial experimental designs and the subsequent analysis of real-time interdependencies of participants may require new approaches.

Also in terms of future experiments, additional brain regions may need to be included into real-time analysis for modulating a main effect. For example, the activation of the SMA or the ipsi-lateral SMA (Figure 3) could provide additional information about modulation of main activation in the contra-lateral SMC since the SMA plays an important role in more complex motor actions such as control of movements or synchronization of both hemispheres^37^,^38^. Thus the SMA could serve as a measure of the degree of synchronicity or complexity of movements. Similar aspects may be important when including the basal ganglia or memory- or emotion-related brain areas (e.g., the activation of the thalamus could serve as a modulating factor resulting from its role in somatosensory circuits, including adjustments of motor activation after positive or negative rewards^39^). The insula provides information about motor control^40^ and self-awareness^41^, while the putamen, being part of nuclei lentiformes, regulates movements^42^ and is involved in learning processes^43^. All of these processes contribute to learning to adjust motor activation in real-time, which could be of special relevance to using real-time fMRI in neurofeedback for rehabilitation or for controlling external devices in handicapped people. Motivation and emotion will always play a very important role for subjects performing in real-time experiments or using brain-machine interfaces.

From a technical point of view, integration of the signals of several brain regions could be accomplished by applying real-time pattern analysis^2^,^6^,^44^. This approach will require a set of calibration factors (one for each region) that will be implemented in future versions of our framework.

In summary, a multi-site rtfMRI environment was implemented and validated with a reliable motor activation paradigm for online control of virtual objects. These settings will be used for subsequent experiments performed with real-time, multi-site social fMRI (such as strategy changes in real-time), thereby enabling many new and interesting experiments related to social and economic issues. Other applications may include joint manipulations of external devices or collaborate actions within a shared virtual reality environment allowing to operate the system as a Hyper-BCI^45^ or to investigate social effects such as synchronization effects between brains^18^.

## Methods

### Subjects

Twelve healthy male adults (25.1 ± 3.1 years, 11 right-handed) participated in this study after giving their written consent. One subject was excluded from data analysis due to incomplete data logging in the main experiment. To avoid cross-gender effects only male volunteers were examined^46^. The study was approved by the local Ethics Committee of the Otto-von-Guericke-University Magdeburg according to the principles of the Declaration of Helsinki.

### Technical Infrastructure

The study was performed on two whole-body MR scanners both equipped with a whole body gradient system (Magnetom 3T Trio; Magnetom 7T; Siemens, Germany). The scanners were located in different buildings belonging to different institutions and different networks. On both scanners, an 8-channel phased-array head coil was used for imaging. To transfer each MR image volume set already during image acquisition to an external computer the original pulse sequence and corresponding image-reconstructing program were modified.

### Implementation

The multi-site-rtfMRI environment is based on a previously described custom-made software^30^ implemented in MATLAB (MATLAB 7.3, MathWorks Inc.). For the present experiment, a 32-bit Microsoft Windows architecture was used. However, the system should be operational on all platforms where MATLAB is running. The software is available on request. To establish a multi-site infrastructure the existing system and the EDL-framework (*Experiment Description Language 1.2*) had to be extended by new modules. These modules handled all information about the levels of the BOLD signals, the scaling factors (to account for different scanner characteristics), analysis of both BOLD signals such as extension of the activated brain region, and location of the stimuli, the timing, and visualization of stimuli on each site. According to the underlying EDL concept, the new experiment control and data analysis parameters can be easily defined in an XML scheme.

To compare activation states of the brains of all participating subjects the EDL-ActivationAnalysis module^30^ was expanded to include a transmitting (OutputModule) and a receiving (ReceiveModule) component. The address of text files to be exchanged was defined as a string (here to define the address of a network drive) but this definition can be easily adapted to include other communication channels or addresses including virtual addresses or internet addresses. Two more modules (cwOutputScan, cwReceiveScan) controlled when the measurements data have to be exchanged. In addition, the type of date to be transmitted (outputParams resp. receiveParams) can be also set. Predefined values at this time are grey-scale values or statistical parameters.

The following small EDL code fragment exemplarily shows the underlying structure for an XML element that specifies the data for mutual information exchange in the according experiment:

<OUTPUTMODULE STATE = “ON” TYPE = “FILE” WRITETIMESTAMP = “ON” MODE = “APPEND”> <LOCATION>[IP]\[PATH]\[FILENAME].TXT</LOCATION> <CWOUTPUTSCAN>10</CWOUTPUTSCAN> <OUTPUTPARAMS OUT_TEMPLATE_ID = “OFF” OUT_TEMPLATE_LABEL = “OFF” OUT_TEMPLATE_ACTLEVEL = “ON” OUT_SOURCE_IMG = “OFF” OUT_MONEY_HUMAN = “ON” OUT_MONEY_OPPONENT = “OFF” OUT_HUMAN_STEPSIZE = “OFF”></OUTPUTPARAMS> </OUTPUTMODULE >

The code fragment describes selected details how the transmission of each partner's results are stored on a central location. The time points when the transfer is occurring have to be defined. Here, these time stamps relate to a time point within a stimulation block and are inserted incrementally. The experimental details of the stimulation run are defined in additional modules (not shown here). The parameters that have to be exported back to each site have also to be defined. In our implementation, these parameters are the current levels of the BOLD signal and the amount of previous rewards of the subject.

The stimulus presentation-module of the original framework had also to be extended in order to be used for stimulus generation for multi-site fMRI. A new module was developed (VisualizeActModule) where type and number of the displayed objects (virtObjects) and the entire time sequence of the stimulation can be defined (randStruct/showScan). This structure allows an easy modification or exchange of the presented stimulus. Thus, each participant can either see only the object manipulated by the own brain activation, or both participants can simultaneously see the objects of both participants. To calibrate the signals of the subjects lying in different MR-scanners scaling factors can be specified (scaleMaxValue). This module also contains algorithms to compare signals of different subjects. More sophisticated methods to analyze and compare the BOLD signals or additional rules to control mutual interactions could be added or implemented here.

**Figure 4:**
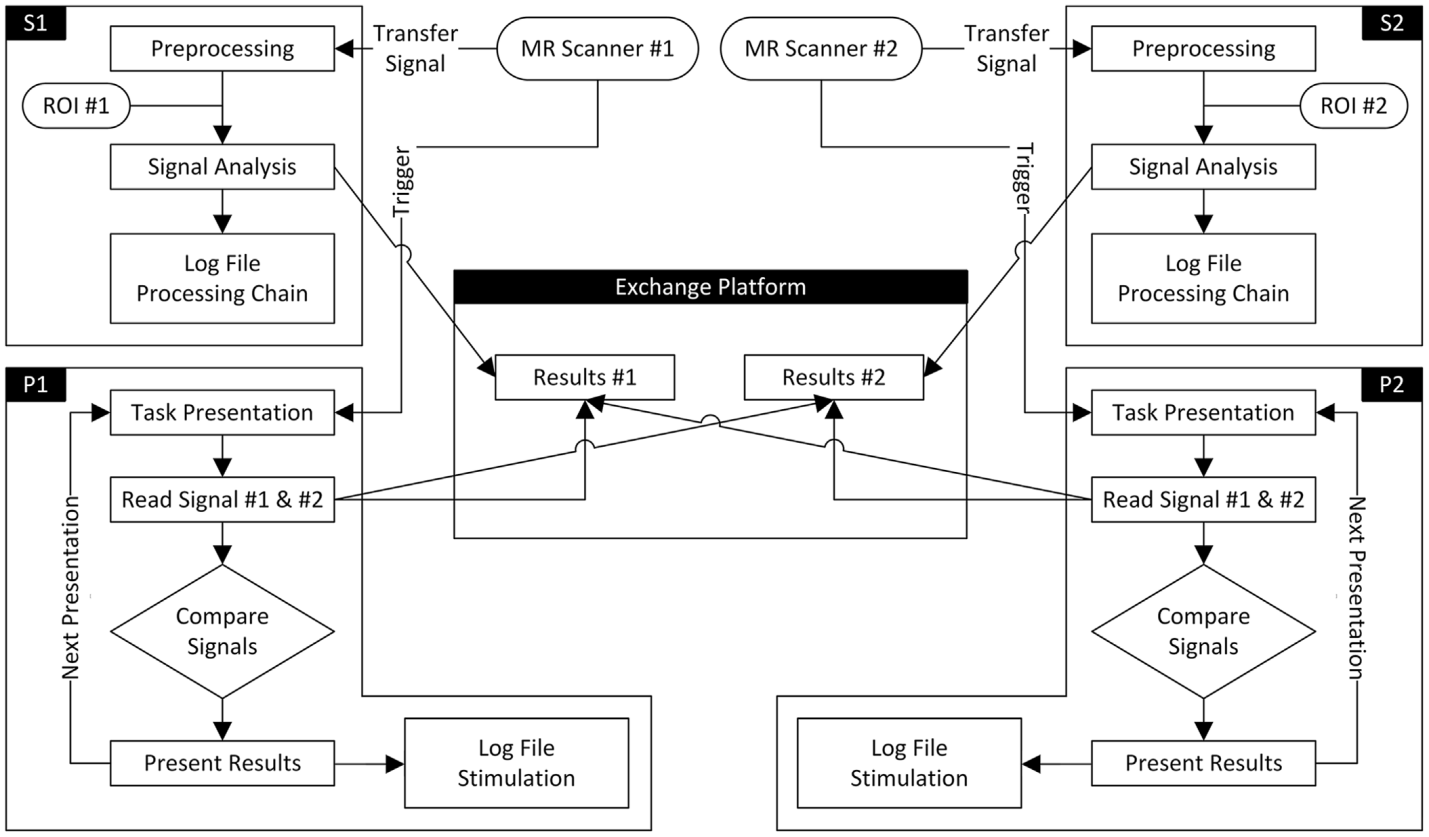
Schematic flow chart of the main processing modules. The data flow starts with the image acquisition at each MR scanner. In each local MR environment a component for pre-processing and statistical analysis (S1 and S2) and a component for signal comparison and presentation (P1 and P2) is installed. Although not used in the validation procedure the system contains a module *Mutual Signal Exchange* (E) where information of each connected site can be exchanged and processed if required. The number of environments can be extended if necessary.

The multi-site rtfMRI framework was implemented here in a first version as two main components (S, P) on both MR scanner environments similar to the concept of hyperscanning^16^. Additionally, a network drive was defined as a central information exchange platform (E) that could be accessed from all involved PCs. After each run, the results of the data analysis were written to a central platform and were thus accessible to other modules (P1 and P2). This allowed to compare the BOLD signals of one or both participants and to generate respective visualizations of the results (Figure 4). All results as well as the task condition and the timing were documented in a stimulus logging module.

### Imaging Protocol

The imaging protocol on both scanners consisted of a high-resolution anatomical T_1_-weighted 3D data set (MPRAGE, 1 mm^3^ isotropic spatial resolution) and gradient-echo EPI for functional imaging with following parameters. 3T: 31 axial slices (no gap), covering the whole brain, repetition time (TR) 2000 ms, echo time (TE) 27 ms, flip angle (FA) 90°, matrix size 64 × 64, 3 × 3 mm^2^ resolution, slice thickness 4 mm). 7T: Limitations imposed by the specific absorption rate (SAR) with the original EPI sequence required to reduce the number of slices to 20 (slice gap 25%) and setting FA to 80°. Therefore, the cerebellum was not scanned at 7T. TE was set to 21 ms due to the shorter T_2_* relaxation at higher field strengths, the other parameters were identical to those at 3T.

### Experimental setup

The experiment was separated into two parts. The first part served to acquire data to define an individual region of interest (ROI) for sensorimotor activation and the individual maximum BOLD signal required for the calibration procedure. For this functional localizer the volunteers performed a sequential finger tapping task (left hand, five blocks of twelve scans each, 2 scans rest, 2 scans finger tapping, 8 scans rest). The activated region was determined with a so-called growing-window or incremental approach^47^ after applying a 3 mm Gaussian kernel to smooth the data. The baseline was determined from run 1 to 3 while the signal was analyzed using a correlation analysis from run 6 to 8 to include the maximum of the hemodynamic response function^30^. The activated brain areas were shown to the experiment supervisor in real-time allowing an immediate quality check. The maximum detected BOLD signal within each individual ROI (which was assumed to reflect the maximum achievable activation) was set to 100%.This maximum signal served as the reference signal for calibrating the signals in the subsequent experiments. After the functional localizer was finished, the selected activated region was stored as a reference ROI for the subsequent experiments.

**Figure 5:**
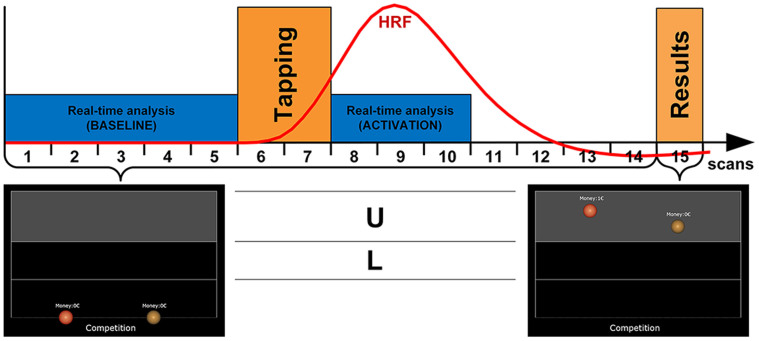
Overview of the experimental setup of the main experiment. Each block consisted of 15 scans. At the beginning of each block, the task to be performed was presented visually on the screen for 10 s (‘single', ‘competition', ‘cooperation'). In the single task only the own sphere was visible thus excluding information exchange between both partners. The next five scans (TR 2 s) were used to determine the current baseline. The sphere had to be moved into the upper (U) or lower (L) part of the field depicted as a light gray rectangle (here, the task required to move the sphere to the upper part). An auditory signal ('start' or 'stop') delivered by earphones started the finger tapping block lasting two scans. To allow the BOLD signal to build up and decay (which was important for a reliable data analysis) a rest period of seven scans followed the finger tapping. Then the spheres moved to the position according to the individual BOLD signal strengths along with the visual presentation whether the task was fulfilled (and paid off) or whether the task was not fulfilled. Thereafter, the presentation was reset and a new round was started.

In the following part (*main* experiment), which was performed immediately after the functional localizer, BCI functionality was applied simultaneously on both sites for each volunteer. The volunteers had to control the location of a virtual object by varying the degree of their motor activation. Two target regions were presented, and the volunteer was instructed to vary the motor activation between a high and a low level in order to place the virtual object in an upper (“U”) or a lower (“L”) location respectively (Figure 5): if the actual activation was within 30% to 60% of the individual maximum activation of the prior functional localizer the current activation was defined as a weak activation (“L”). To avoid a strategy where the subjects would just stop finger tapping when asked to reproduce the low motor activation, mean activation levels below 30% were rated as insufficient, and the task was defined as not fulfilled. If more than 60% of the maximum activation was reached, current activation was defined as a strong activation (“U”). Using these relative measures, different signal strengths of two scanners and individual differences could be taken into account.

The main experiment consisted of three tasks to be performed in a block design (see Fig. 5). Tasks were distributed in a random order with a total of 20 runs for each task. Both target regions had to be reached a total of 30 times, i.e., ten times for each task (‘single', ‘cooperation', ‘competition'). The baseline was determined from the first five scans before start of finger tapping. The real-time analysis of the BOLD signal was performed for 6 sec, starting immediately after finger tapping thus encompassing the main parts of the positive BOLD signal. After the rest phase, the result was displayed for 2 sec. The current activation level was determined using a sliding-window technique encompassing only the actual baseline and the BOLD signal^48^.

To determine which brain networks were activated in each individual when controlling the sensorimotor activation, each subject had to reach the current target region independent of the activation of the other participant (‘single' task). Accordingly, each volunteer saw only his own sphere. A reward was assigned when the subject reached the required activation strength determined by the target position of the sphere. In two more tasks, the reward depended additionally on the activation of the partner (social tasks). In the “cooperation” task the reward was assigned only to each participant when both reached the same position (in the upper or lower part, similar to the single task) while in the “competition” task the partner who reached the requested position most accurately received the reward.

The focus of this study is validation of the calibration procedure which uses only the BOLD signals acquired during the ‘single' condition. The signals serve as a reference for the capability for each individual when controlling an object by adapting the activation of the motor cortex as additional effects due to mutual information flow or interpersonal dependencies are excluded. The social tasks will introduce additional features in brain activation patterns, which will be discussed in a forthcoming publication.

The average time for reconstruction and transmission of data to each of the statistics computers (S1, S2) was 467 ms ± 39 ms. The average response time of the internet connection was below 1 ms. During a TR of 2000 ms only the few values representing the mean activation of the selected ROI but not the whole image data set had to be transmitted. Average computation time for data processing was 1447 ms ± 226 ms which was also well within one TR (2000 ms).

### Offline data Analysis

To check which brain regions were activated in addition to the contralateral sensorimotor cortex used for online analysis, an offline analysis of both the *calibration* and *main* experiment was performed using BrainVoyager QX 2.4.1^49^. The analysis included slice scan-time correction, 3D motion correction and a spatial smoothing with a 3 mm FWHM Gaussian kernel. Functional images were co-registered to the anatomical volume data set, and normalized into Talairach space using the standard landmark method^50^. Activation was estimated by convolving the vectors of onset with the canonical HRF. The resulting general linear model (GLM) was corrected for serial correlation using a second-order autoregressive model AR(2). For each subject, first-level analyses were performed for the weak and strong tapping condition. In addition, a random effects analysis (RFX) of the group data (collapsed over both tapping conditions) was applied.

To determine whether there was a significant difference between cortical activation in the weak and the strong tapping conditions, the BOLD signals of clusters within the SMC were extracted offline using the MATLAB toolbox NeuroElf 0.9c (Jochen Weber, http://www.neuroelf.net, accessed 10/13/2014). For comparison with the real-time data analysis, BOLD signals of scan 8 to 10 of each block were averaged and used for further statistical analysis as here the BOLD signal was expected to exhibit its maximum amplitude (Figure 5, blue activation block). The difference between weak and strong tapping was estimated using a mixed model to allow for the correlation of the data. All statistical analyses were conducted using SAS (SAS/STAT® 9.2, SAS Institute Inc.).

## Author Contributions

S.B. prepared the manuscript including all figures/tables and was responsible for the data collection and analysis. R.L., J.M., M.L. and J.B. were responsible for manuscript preparation and data analysis. C.T. and J.S. were responsible for the technical part of the data collection. All authors reviewed the manuscript.

## Figures and Tables

**Table 1:** Results of the random effects group analysis for the single tapping condition. Talairach coordinates (X, Y, Z) of cluster center, cluster sizes (c) and the mean t-value (T) of each cluster (subject 1 was excluded, for details see text)

anatomic region	3T [p < 0.005, FDR corrected]					7T [p < 0.005, FDR corrected]				
	coordinates			c	T	coordinates			c	T
	X	Y	Z			X	Y	Z		
RH SMC	38	−26	50	(80)	8.47	38	−27	50	(227)	9.09
LH SMC	-					−35	−16	57	(38)	7.93
SMA	1	−2	48	(98)	9.09	2	−13	47	(88)	7.80
LH Putamen	−26	−1	8	(115)	9.13	−27	0	10	(110)	7.75
RH Putamen	25	−3	6	(114)	8.37	30	−7	9	(93)	8.86
LH Thalamus	−17	−13	16	(8)	9.86	−13	−16	6	(24)	8.29
RH Thalamus	11	−5	15	(5)	6.59	15	−16	7	(35)	8.68
